# Mapping the intersection of sudden cardiac death and COVID-19: a comprehensive bibliometric analysis (2020–2024)

**DOI:** 10.3389/fcvm.2024.1472337

**Published:** 2024-11-18

**Authors:** Sudip Bhattacharya, Alok Singh, Akanksha Singh, Sukhpreet Singh

**Affiliations:** ^1^ Department of Community and Family Medicine, All India Institute of Medical Sciences, Deoghar, India; ^2^ Faculty of Naturopathy & Yogic Sciences, SGT University, Gurugram, India; ^3^ Department of Community Medicine, Faculty of Medicine and Health Sciences, SGT University, Gurugram, India; ^4^ Mahatma Gandhi Kashi Vidyapith, Varanasi, India; ^5^ Department of Health, Haryana Civil Medical Services (HCMS), Panchkula, India

**Keywords:** COVID-19, sudden cardiac death, SARS-CoV-2, pandemic (COVID-19), cardiac arrest (CA)

## Abstract

**Introduction:**

A direct causal link between Sudden Cardiac Death (SCD) and COVID-19 is unproven, although current data suggest a plausible association.

**Aim:**

This study aims to map and analyze the intersection of research on sudden cardiac death and COVID-19 through a comprehensive bibliometric analysis.

**Methodology:**

This study searched the literature by applying the keywords “COVID-19” AND “Sudden Cardiac Death (SCD)”. Literature retrieved using the above keywords and published were included with a time limit from 1st January 2020 to 8th Aug 2024.

**Results:**

The bibliometric analysis of COVID-19 and Sudden Cardiac Death highlights key research trends from 2020 to 2024, revealing a rapid surge in scientific output during the pandemic. 2,915 articles were retrieved, with 70.5% being original research, reflecting a strong focus on new empirical evidence. The peak in publications occurred in 2021, driven by the urgent need to understand the cardiovascular implications of COVID-19. However, a decline in publications and citations in subsequent years suggests a shift in research priorities and a potential saturation in initial research areas. Leading institutions like Harvard Medical School, Mayo Clinic, and the University of Pennsylvania played a significant role, with the U.S., China, and the U.K. being top contributors. Despite fewer publications, China's research had a notable impact, indicated by high average citations per article. Keyword analysis identified “COVID-19” and “SARS-CoV-2” as dominant themes, with related terms like myocarditis and heart failure reflecting major cardiovascular concerns. Co-citation analysis revealed seminal works that shaped the discourse, with influential articles by Xu Z et al. and Guo T et al. frequently cited. The collaborative nature of research, especially among top institutions and countries like the U.S., Italy, and the U.K., was evident in network analyses. This study provides a comprehensive overview of the research landscape, highlighting significant contributions, emerging themes, and future research directions in understanding and mitigating the cardiovascular impacts of COVID-19.

## 1 Introduction

A direct causal link between Sudden Cardiac Death (SCD) and COVID-19 is unproven, current data suggest a plausible association (1). An increased incidence of SCD was reported in both community and hospital settings during the pandemic (1). In Houston, cardiac arrest calls resulting in death rose by 45%. Italy reported a 58% rise in Out-of-Hospital Cardiac Arrests (OHCA) compared to the previous year, with 77.4% of the increase linked to COVID-19 (2). Paris also saw a two-fold rise in OHCA. These increases may be due to both direct COVID-19 deaths and indirect effects like lockdowns and healthcare system issues. Chinese hospital data showed that 27.8% of admitted COVID-19 patients had a myocardial injury, with higher mortality rates in those with elevated troponin levels. A study in Philadelphia reported a 1.3% incidence of cardiac arrest among 700 COVID-19 patients, all in the ICU (3). A global survey by the Heart Rhythm Society found atrial fibrillation (21%) to be the most common tachyarrhythmia among hospitalized COVID-19 patients, with severe bradycardia and complete heart block each at 8% (4). The survey also reported a 4.8% incidence of ventricular tachycardia/ventricular fibrillation arrest and a 5.6% incidence of pulseless electrical activity, highlighting a potential link between COVID-19 and increased SCD risk (1).

Pre-existing chronic medical conditions, especially cardiovascular diseases (CVDs), may be at heightened risk for adverse outcomes due to COVID-19. The interaction between COVID-19 and pre-existing heart conditions such as dilated cardiomyopathy (DCM), ischemic cardiomyopathy, hypertrophic cardiomyopathy (HCM), and various channelopathies is critical, as these patients may experience more severe illness and complications. Dilated Cardiomyopathy (DCM) involves the weakening of the heart muscle, impairing its ability to pump blood efficiently. In the context of COVID-19, viral infection and subsequent inflammatory responses can exacerbate heart failure symptoms in DCM patients (5). The added strain from COVID-19-related lung involvement and systemic inflammation can worsen heart function, leading to increased morbidity and mortality. Similarly, ischemic cardiomyopathy, caused by reduced blood flow due to coronary artery disease, can be severely impacted during COVID-19 infections. As discussed in the literature, the virus's pro-inflammatory state may aggravate pre-existing ischemia, potentially leading to acute coronary events or worsening heart failure (6).

Patients with ischemic cardiomyopathy often have limited cardiovascular reserves, and the systemic inflammation induced by COVID-19 may trigger arrhythmias, myocardial injury, or even sudden cardiac death. Hypertrophic cardiomyopathy (HCM) is a genetic disorder characterized by abnormal heart muscle thickening.COVID-19 may disproportionately affect HCM patients due to their predisposition to arrhythmias, such as atrial fibrillation and ventricular tachycardia. Additionally, the risk of thromboembolic events, already higher in HCM, could be further elevated due to COVID-19's known association with coagulopathy (7). Channelopathies**,** which affect the heart's electrical signalling, may also pose a significant risk during COVID-19 infections, as highlighted in the literature (8). These conditions, such as Long QT syndrome and Brugada syndrome, can lead to dangerous arrhythmias, especially when combined with the systemic stress of an infection. Medications commonly used in COVID-19 treatment, like antiviral agents or anti-inflammatory drugs, may further prolong the Q.T. interval, exacerbating the risk of fatal arrhythmias (8).

Apart from that, cardiorespiratory fitness, partly influenced by regular physical activity, is a strong predictor of mortality and adverse health outcomes. Research shows that higher fitness levels reduce the risk of non-communicable diseases like cardiovascular disease, diabetes, and certain cancers. This is likely due to the way cardiorespiratory fitness reflects the health of various bodily systems, extending beyond traditional risk factors (9). Regular moderate physical activity also lowers the risk of respiratory infections and enhances vaccine responses through immunomodulatory effects. Additionally, muscle contractions release immune mediators like IL-6, driving anti-inflammatory effects that benefit conditions such as cancer, cardiovascular disease, diabetes, and cognitive impairment (9).

## 2 Aim

This study aims to map and analyse the intersection of research on sudden cardiac death and COVID-19 through a comprehensive bibliometric analysis.

## 3 Materials and methods

### 3.1 Data source

The online SCOPUS database was used for data retrieval since it is the most commonly used database in medical research.

### 3.2 Search strategy

**Figure 1:**
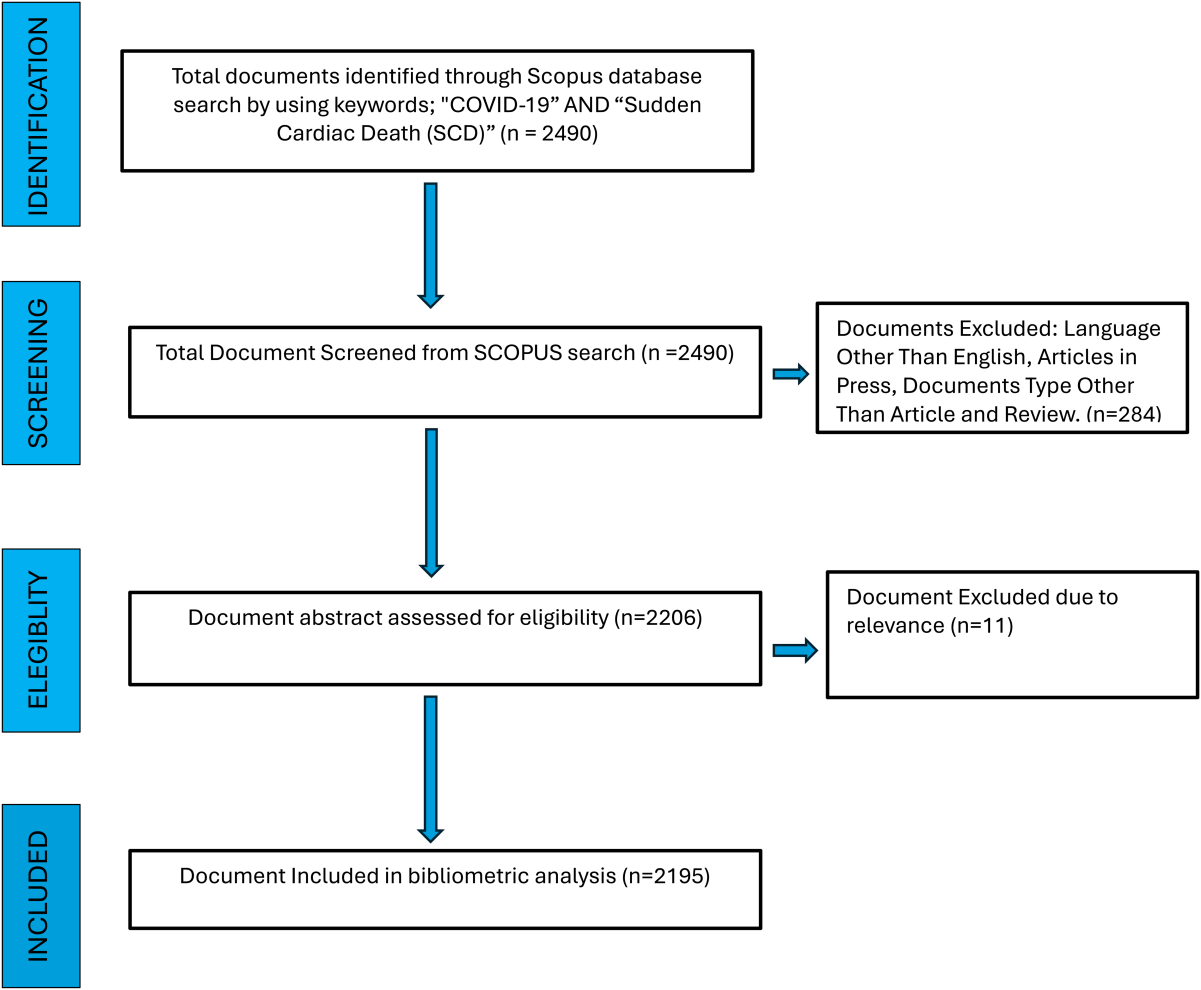
PRISMA guidelines.

This study searched the literature by applying the keywords “COVID-19” AND “Sudden Cardiac Death (SCD)”. Literature retrieved using the above keywords and published were included with a time limit from 1st January 2020 to 8th August 2024 (Figure 1).

### 3.3 Analysis

All the references and records of the search results were exported in CSV format. For literature related to COVID-19 and Sudden Cardiac Death (SCD), the number and types of documents, countries, and institutions were analyzed, and the relationship between countries, institutions, and authors was also analyzed. Analysis of related journals in which the searched literature was published was also carried out. Keywords used in this literature were also analyzed. Data collected were later imported into Biblioshiny (R version 4.2.2; Institute for Statistics and Mathematics, Vienna, Austria; www.r-project.org) and Microsoft Office Excel 2021 (Redmond, WA, USA) for further analysis. For the visualization and the analysis of sources, authors, and documents, Biblioshiny is used (10). Different bibliometric indicators are analyzed through Biblioshiny to assess the output of countries, authors, institutions, and journals. The number of articles is used for the evaluation of the output of countries, authors, institutions, and journals the number of articles is used. The number of articles is also used to assess productivity (11). Impact in the academic community is indicated by total citations, while local citations are used to assess impact in specific fields (12). They are the three main dimensions for assessing the quality of research. The h-index, where the scholar has published h papers that have each been cited at least h times, is an indicator that combines productivity and impact (13). In addition, three field plots, country/institutional collaboration plots, and affiliation/authors’ production over time were also plotted using Biblioshiny.

## 4 Results

### 4.1 Analysis of trends of publication and research

**Figure 2:**
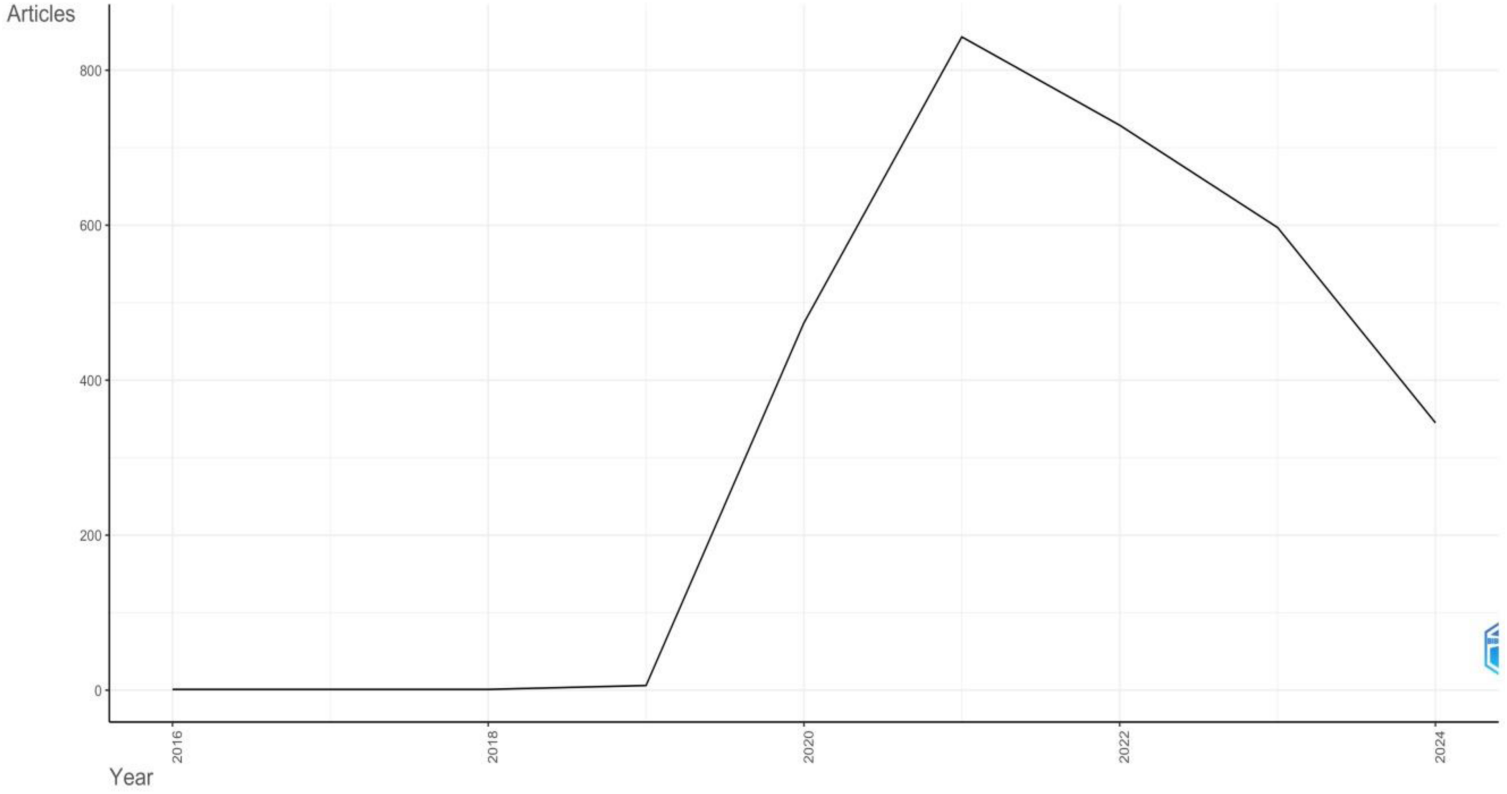
Annual scientific production.

From 1st January 2020 to 8th August 2024, a total of 2,915 articles (Figure 1) with the subject terms “**COVID-19**” and “**Sudden Cardiac Death**” were retrieved from the SCOPUS, which consisted of the following article types: Article 2,056 (70.5%); Review 859 (25.9%). Figure 2 reflects the trend in research on COVID-19 and SCD. It illustrates the annual variations in scientific output in the aforementioned research areas over this period. The analysis indicates that global scientific publications on COVID-19 and SCD peaked in 2021, with 842 publications, marking it as the most productive year. Consequently, there has been a notable increase in the volume of international academic literature in the last three years (2020 to 2023) compared to previous years before 2020, suggesting heightened research interest in these topics since 2020. In 2020, the average number of citations per article was 16.9. In 2021, it was 5.3. In 2022, it was 3.9. In the year 2023, it was 4. In 2024, the average citation per article is 0.9.

### 4.2 Analysis of most relevant authors

**Figure 3:**
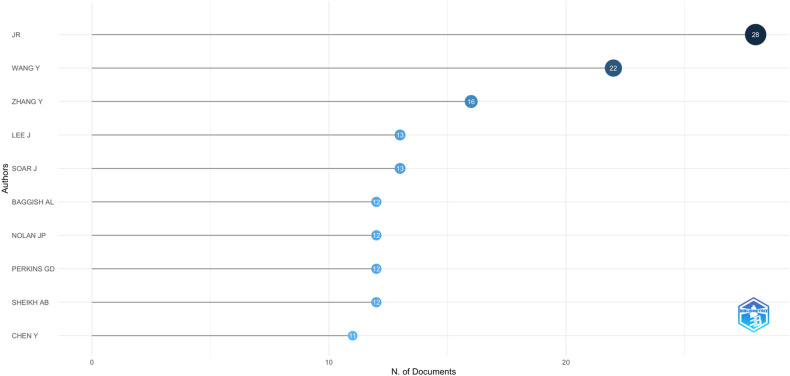
Most relevant authors.

The current paragraph emphasizes the leading researchers in the fields of COVID-19 and SCD, focusing on their publication volume and the impact of their work. Figure 3 illustrates the ten most prolific authors and the number of publications each has contributed to this area.

### 4.3 Analysis of the most relevant organizations

**Figure 4:**
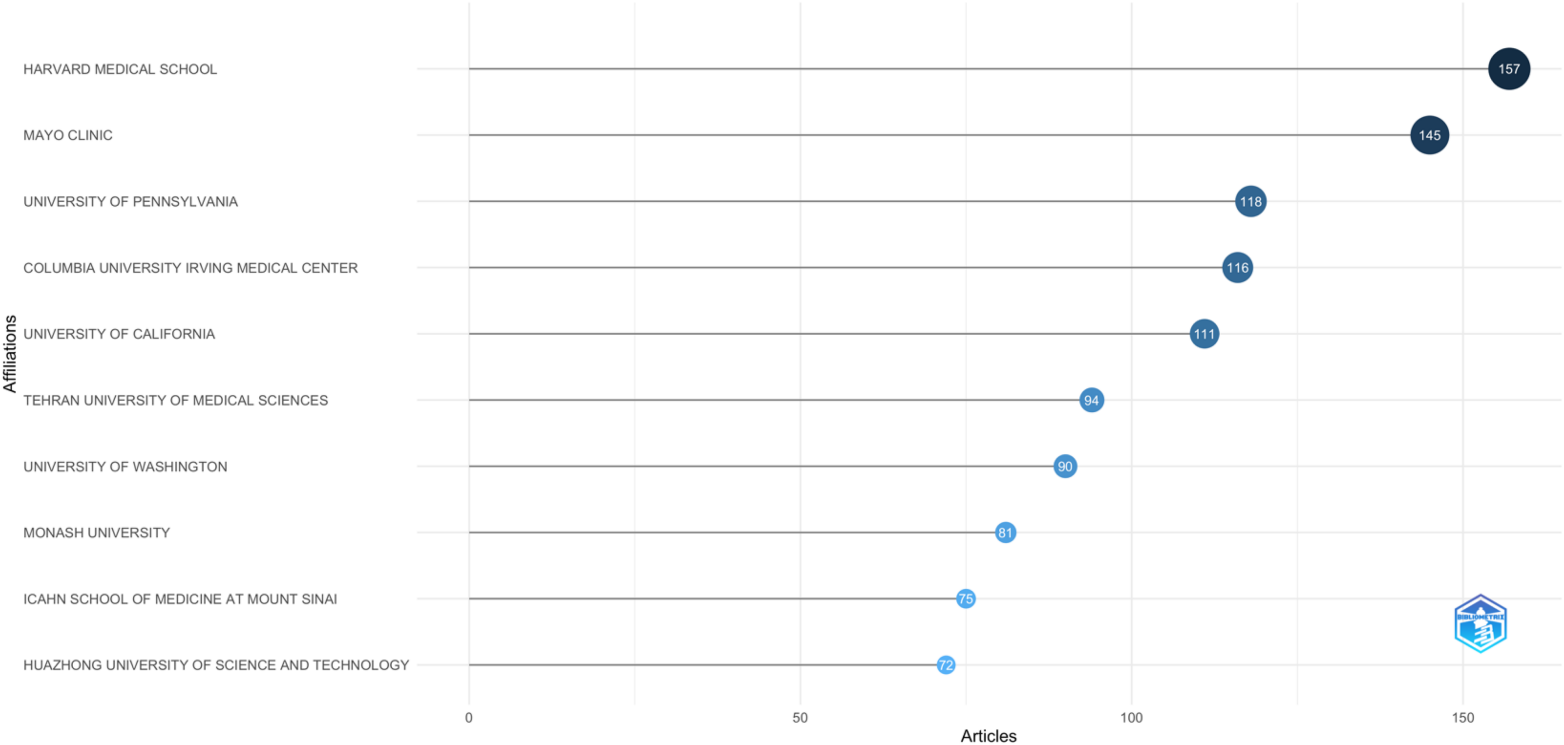
Most relevant affiliations.

**Figure 5:**
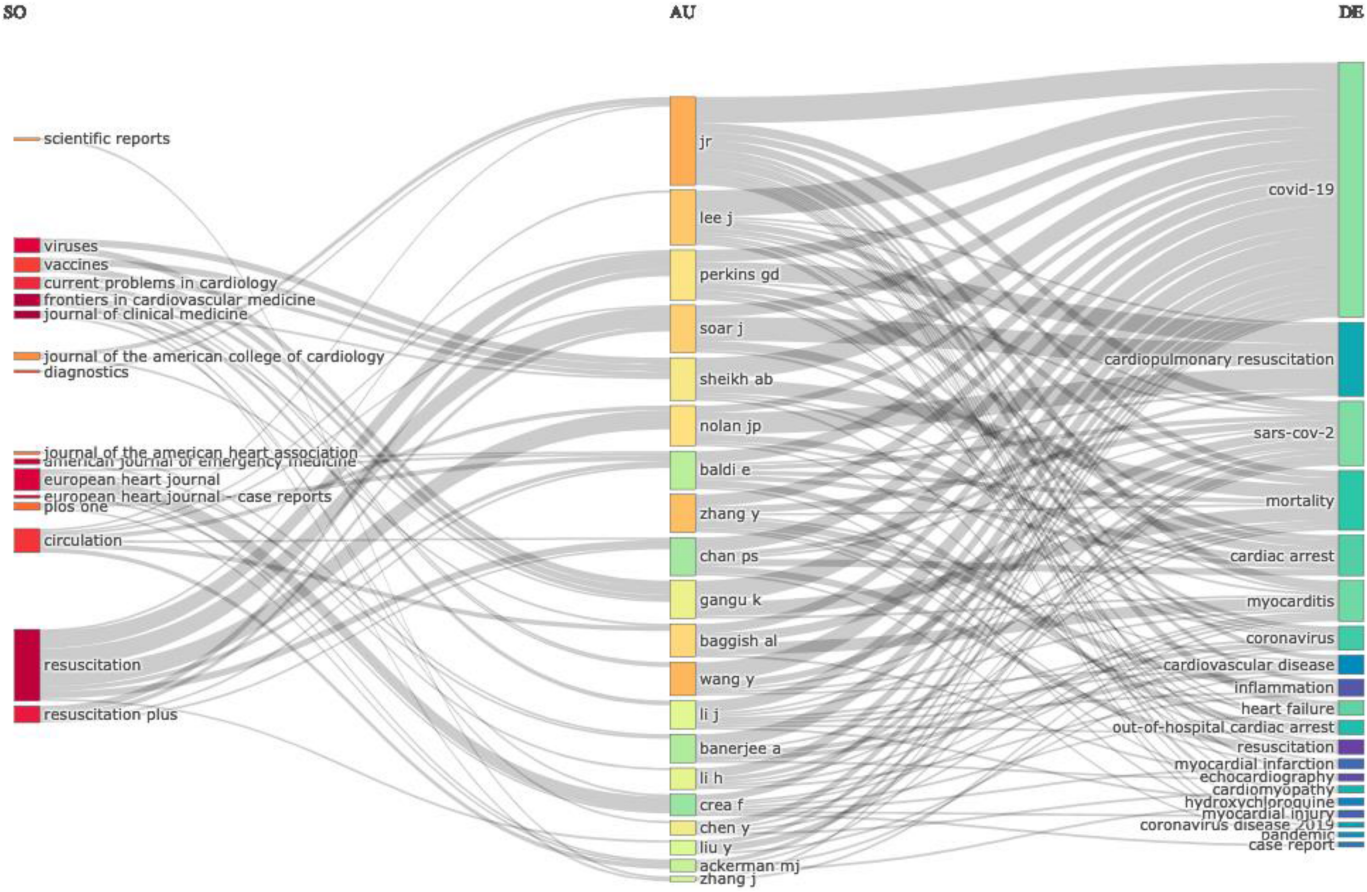
Three-field plot diagram.

Figure 4 showcases the top ten organizations in research on COVID-19 and SCD. Harvard Medical School leads with 157 publications, followed by Mayo Clinic with 145 publications. The University of Pennsylvania ranks third with 118 publications, while Columbia University Irving Medical Center has 116 publications. The University of California completes the top five with 110 publications. Figure 5 Uses a three-field plot diagram to illustrate the pattern of authors’ publications in different related topics and journals.

### 4.4 Analysis of the country's scientific production

Table 1 highlights the top 10 countries contributing to COVID-19 and SCD research, providing insights into the total number of publications, overall citations, and average citations per article. The data reveals that only three countries—the USA, China, and the United Kingdom—have produced over a hundred publications in these areas from 2020 to 2024. The USA leads with 831 publications and 26391citations, making it the most influential contributor. China has 169 publications and 12,568 citations, while the United Kingdom has 108 publications and 4,868 citations. Notably, despite China's third-place ranking in both total publications and citations, it excels in average citations per article, surpassing the other countries.

**Table 1:** Country's scientific production.

Country	T.C.	Average article citations	Articles
USA	26,391	31.80	831
China	12,568	74.40	169
United Kingdom	4,868	45.10	108
Germany	3,400	34.70	98
Italy	3,346	14.90	225
France	2,725	39.50	69
Iran	1,204	15.10	80
Canada	1,196	16.20	74
Spain	996	13.10	76
Australia	907	17.10	53

### 4.5 Analysis of most preferred periodicals

**Figure 6:**
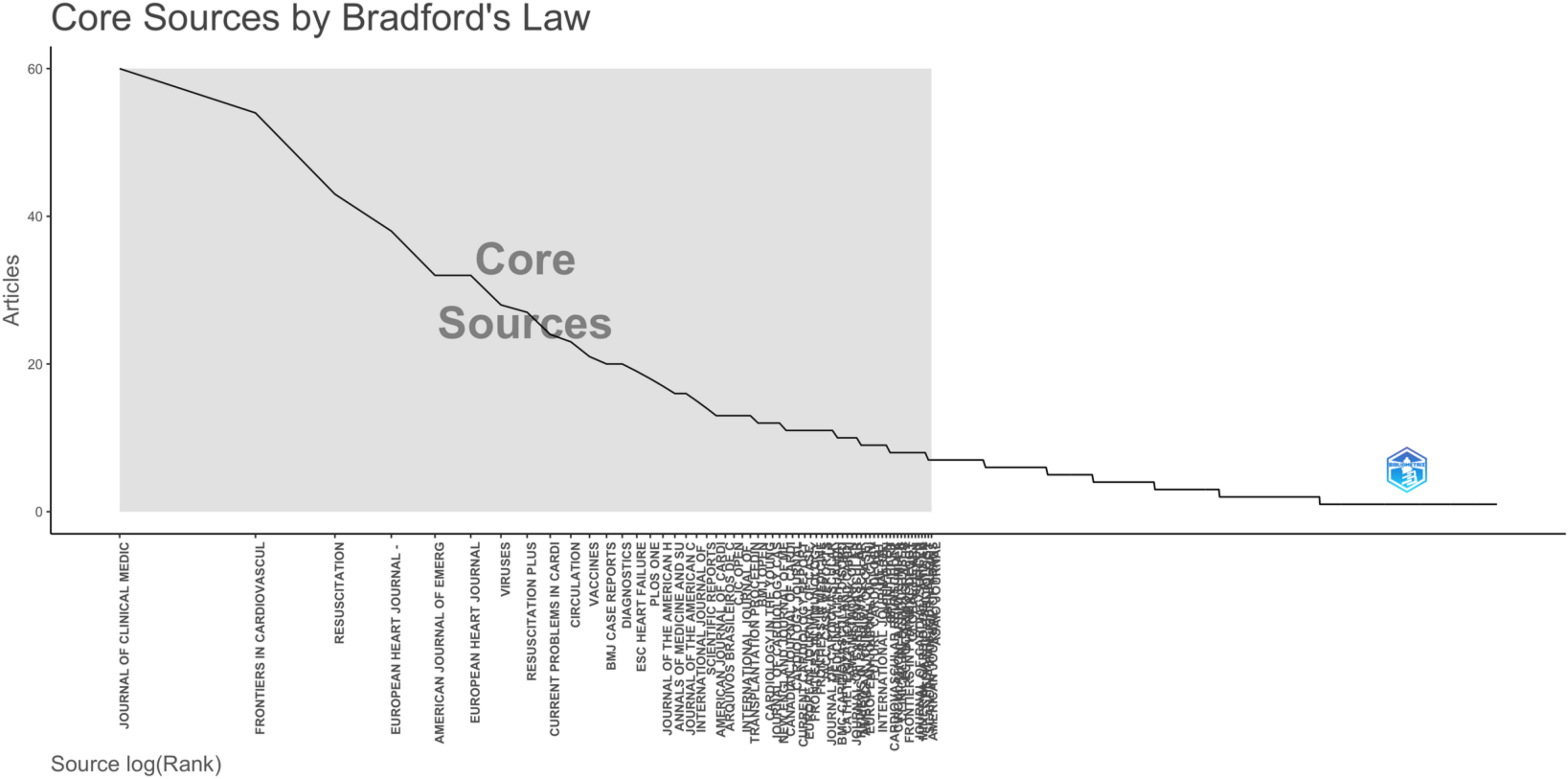
Bradford law.

In Bradford's law, the core sources, or the nucleus of journals, are those that focus intensely on a specific research area. Figure 6 illustrates that the top ten journals, detailed in Table 2, account for approximately one-third of the total documents in the collection. These leading journals are the primary venues for publishing articles in our bibliographic collection. Notably, the Journal of Clinical Medicine tops the list with 60 articles, making it the most frequently selected publication venue. It is followed by Frontiers in Cardiovascular Medicine and Resuscitation, which have published 54 and 43 articles, respectively.

**Table 2:** Sources and their contributions.

Source	Articles
Journal Of Clinical Medicine	60
Frontiers In Cardiovascular Medicine	54
Resuscitation	43
European Heart Journal—Case Reports	38
American Journal Of Emergency Medicine	32
European Heart Journal	32
Viruses	28
Resuscitation Plus	27
Current Problems In Cardiology	24
Circulation	23

### 4.6 Analysis of highly cited research publications

Table 3 highlights the top ten most frequently cited research publications on COVID-19 and Sudden cardiac death, spanning from 2020 to 2024. Leading this list is the article “Pathological Findings of COVID-19 Associated with Acute Respiratory Distress Syndrome”, authored by Xu Z et al. and published in The Lancet Respiratory Medicine, which has amassed an impressive 6,500 citations (14). Following closely is “Cardiovascular Implications of Fatal Outcomes of Patients with Coronavirus Disease 2019 (COVID-19)” by Guo T et al., featured in JAMA Cardiology, with 2,940 citations (15).

**Table 3:** Top ten most frequently cited research publications.

Paper	DOI	Total citation
XU Z, 2020, LANCET RESPIR MED	10.1016/S2213-2600 (20)30076-X	6,500
GUO T, 2020, JAMA CARDIOL	10.1001/jamacardio.2020.1017	2,940
VADUGANATHAN M, 2020, NEW ENGL J MED	10.1056/NEJMsr2005760	1,610
PUNTMANN VO, 2020, JAMA CARDIOL	10.1001/jamacardio.2020.3557	1,547
DRIGGIN E, 2020, J AM COLL CARDIOL	10.1016/j.jacc.2020.03.031	1,484
TSAO CW, 2023, CIRCULATION	10.1161/CIR.0000000000001123	1,432
XIE Y, 2022, NAT MED	10.1038/s41591-022-01689-3	1,019
GUZIK TJ, 2020, CARDIOVASC RES	10.1093/cvr/cvaa106	1,012
NISHIGA M, 2020, NAT REV CARDIOL	10.1038/s41569-020-0413-9	918
BELHADJER Z, 2020, CIRCULATION	10.1161/CIRCULATIONAHA.120.048360	906

Another significant contribution is “Renin–Angiotensin–Aldosterone System Inhibitors in Patients with COVID-19” by Vaduganathan M et al., published in The New England Journal of Medicine, which has received 1,610 citations (16). Lastly, Puntmann VO et al.'s article, “Outcomes of Cardiovascular Magnetic Resonance Imaging in Patients Recently Recovered from Coronavirus Disease 2019 (COVID-19),” has garnered 1,547 citations (17). These publications are recognized as the most influential in the field, reflecting their substantial impact on research concerning COVID-19 and Sudden Cardiac Death.

### 4.7 Analysis of authors keywords

**Figure 7:**
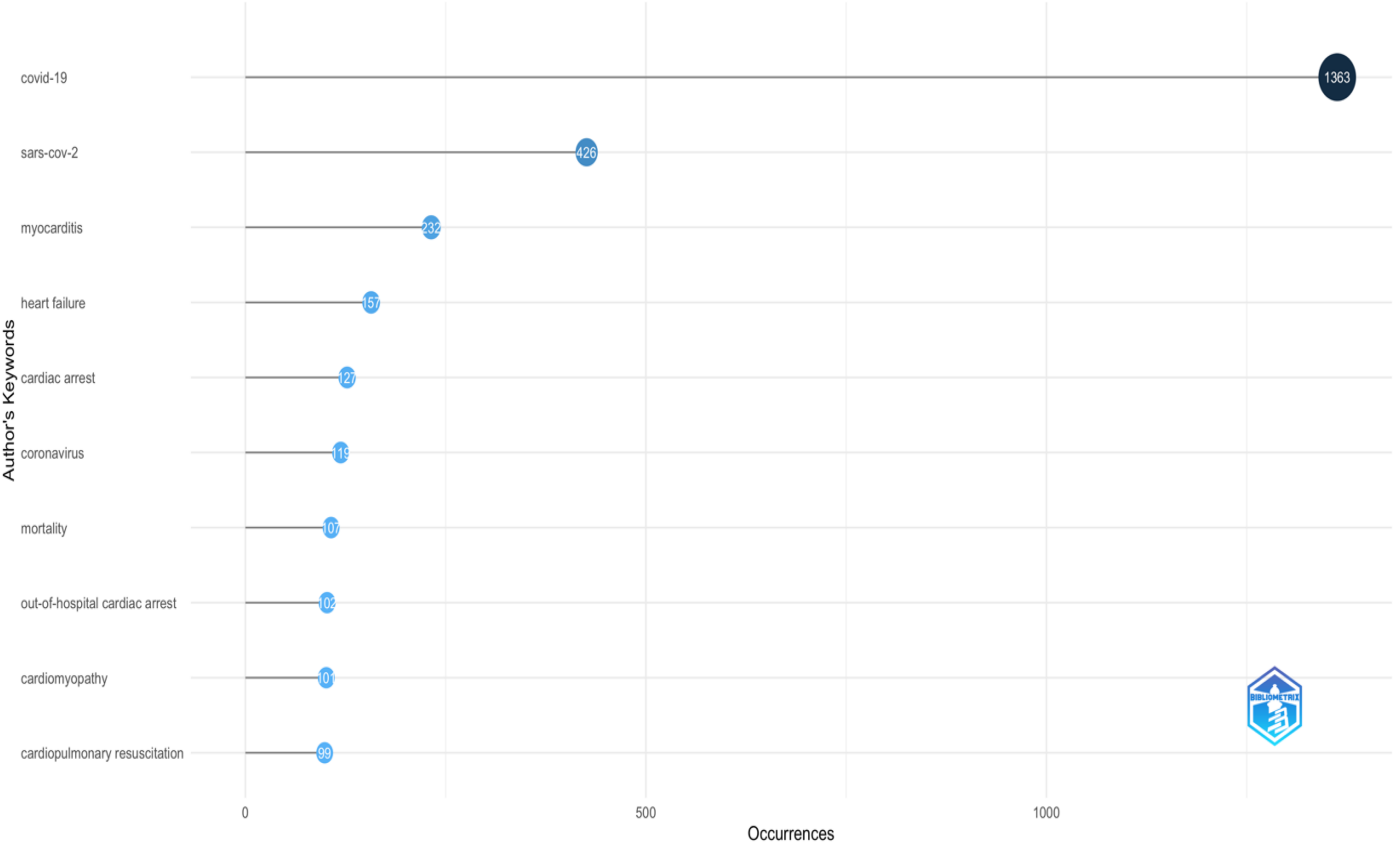
Most relevant words.

**Figure 8:**
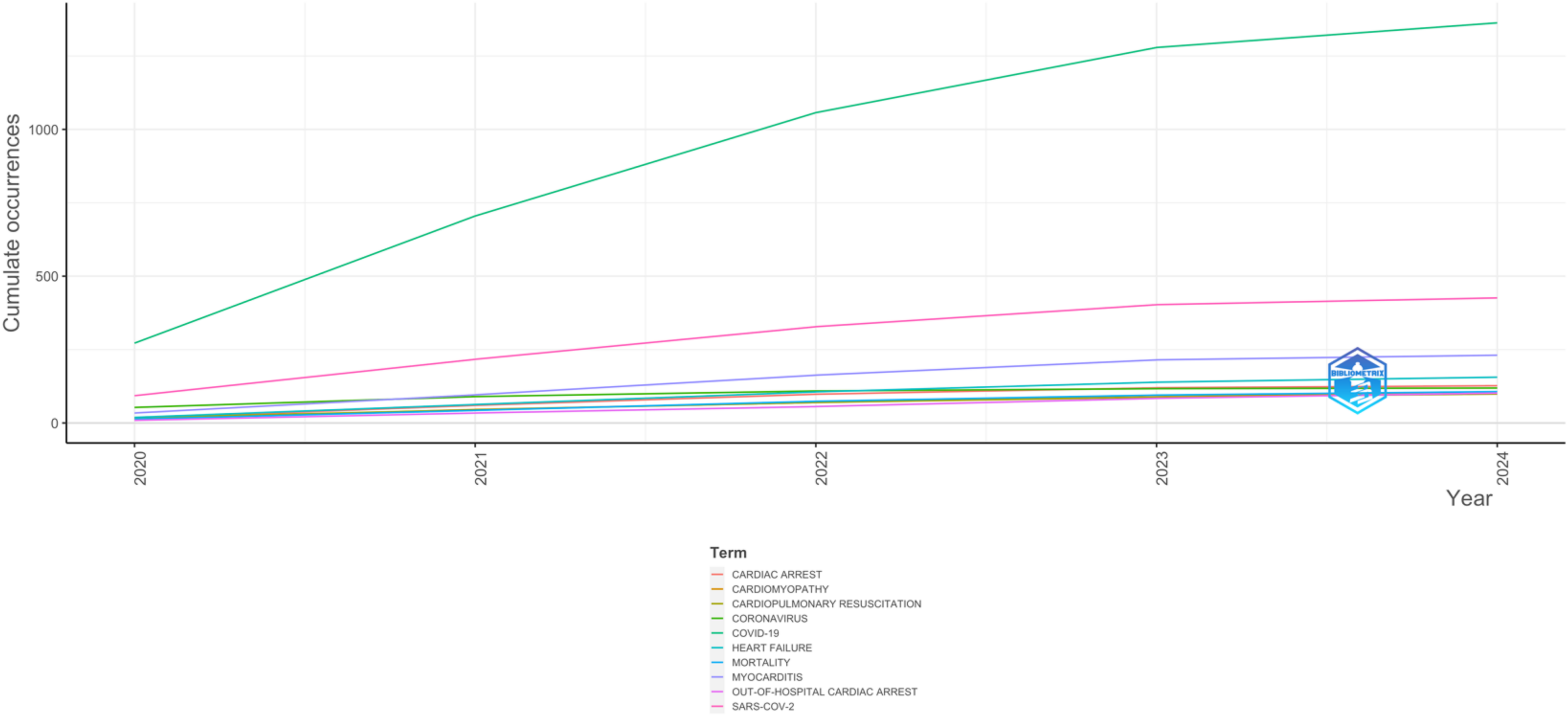
Words frequency over time.

**Figure 9:**
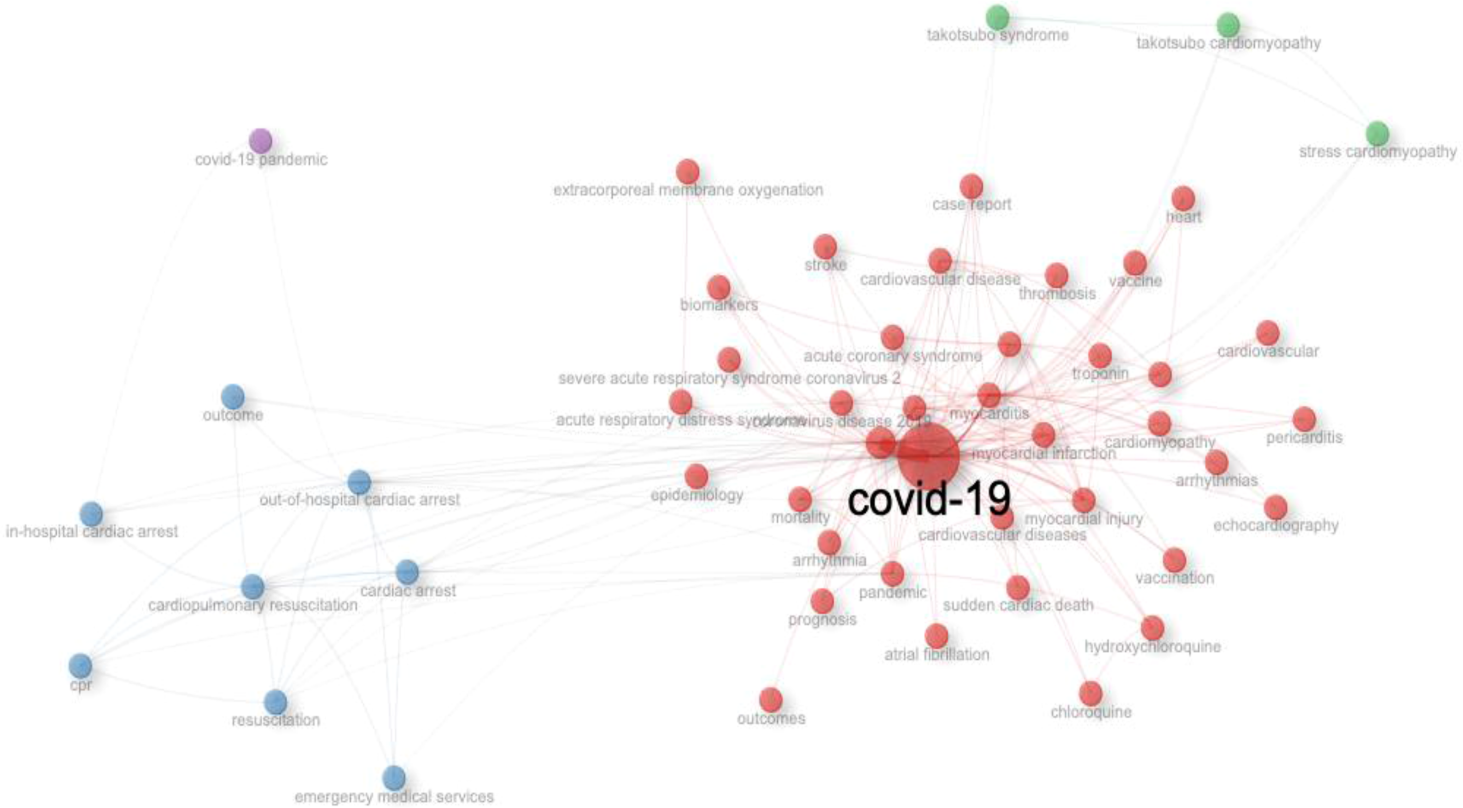
Co-occurrence network.

In this section, we utilize keyword analysis and co-occurrence techniques to examine research trends and advancements in COVID-19 and Sudden Cardiac Death, identifying gaps in the literature and potential future research directions. Figure 7 highlights the top fifteen keywords, with “Covid-19” appearing most frequently (1,363 times), followed by SARS-CoV-2 (426), myocarditis (232), heart failure (157), and cardiac arrest (127). The relationship between COVID-19 and Sudden Cardiac Death research is illustrated in the word growth graph in Figure 8. This graph shows the annual increase in the main keywords related to various cardiac events alongside COVID-19, such as cardiac arrest, cardiomyopathy, cardiopulmonary resuscitation, coronavirus, heart failure, myocarditis, out-of-hospital cardiac arrest, and SARS-CoV-2. Notably, from 2020 to 2024, there has been a significant rise in the cumulative occurrences of these keywords: mortality (107), cardiac arrest (127), cardiomyopathy (101), cardiopulmonary resuscitation (99), coronavirus (119), heart failure (156), myocarditis (231), out-of-hospital cardiac arrest (102), and SARS-CoV-2 (426), with Covid-19 leading at 1,363 occurrences. Additionally, we analyzed the co-occurrence of authors’ keywords using biblioshiny to understand the key topics and the evolution of issues in this research field. Figure 9 presents the results of the co-occurrence network study, where the size of each node (keyword) indicates the number of occurrences (18). Covid-19, as the largest node, is confirmed as the most frequent keyword. Figure 9 further illustrates that the keywords with the highest betweenness after COVID-19 include SARS-CoV-2, Vaccination, mortality, trophonin, biomarkers and cardiovascular events like myocarditis, out-of-hospital cardiac arrest, cardiac arrest, arrhythemia, cardiovascular disease, acute coronary syndrome, coronavirus, Takotsubo syndrome, and sudden cardiac death.

### 4.8 Keyword evolution trend

**Figure 10:**
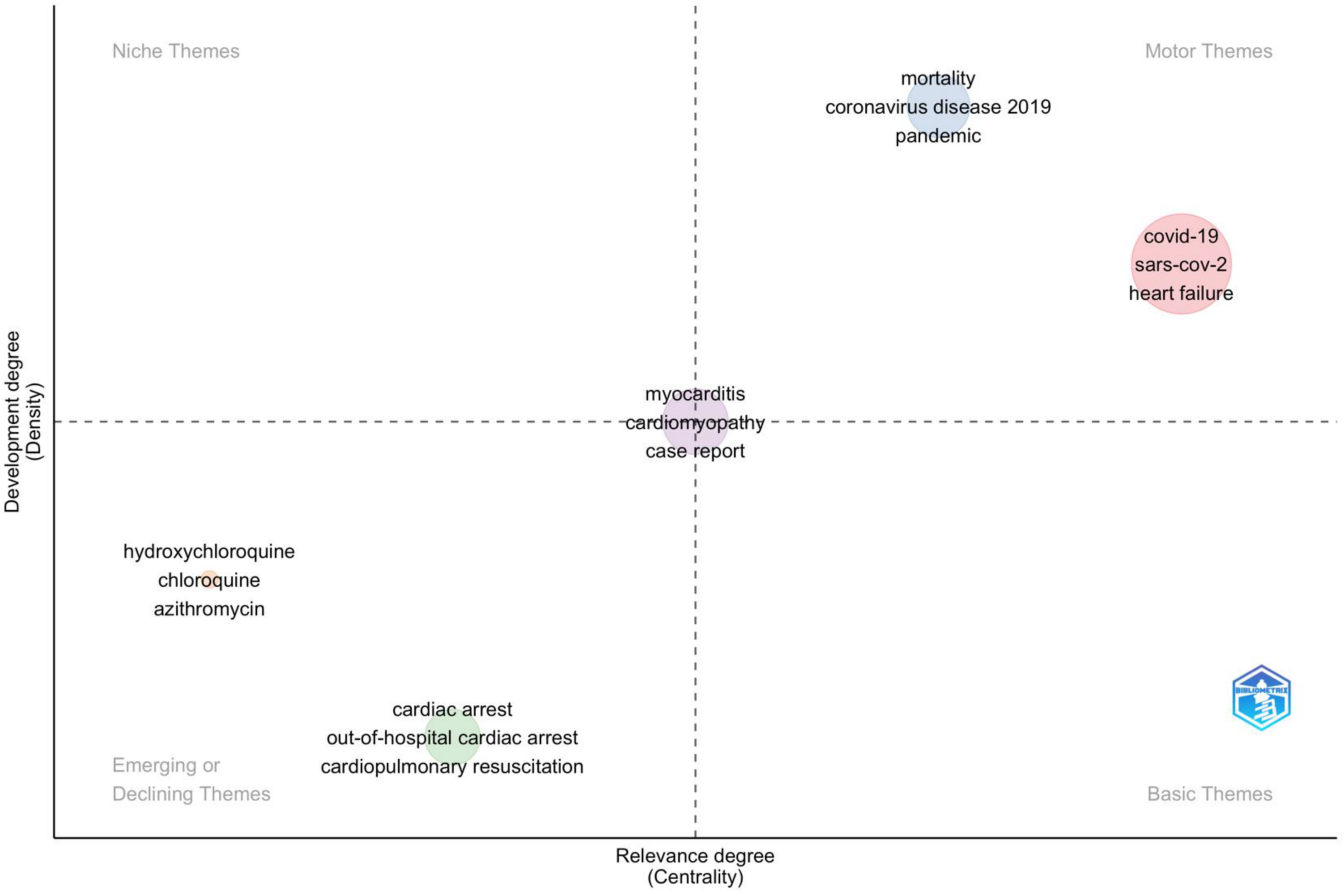
Thematic map.

Utilizing a clustering algorithm on the keyword network enables the identification of various themes within a specific domain. These clusters or themes can be visualized on a strategic or thematic map. In this map, each bubble represents a network cluster, with the bubble's name being the word with the highest occurrence in the cluster. The bubble's size reflects the frequency of the cluster's word occurrences, and its position is determined by the cluster's centrality and density metrics. Centrality indicates the theme's importance within the entire research field, while density measures the theme's development (19). Thematic maps were thus created to illustrate the evolution of keyword trends, as depicted in Figure 10. This map is divided into four quadrants, which include the upper right quadrant, which highlights motor themes, which are well-developed themes related to Sudden cardiac Death and COVID-19.This includes COVID-19, SARS-CoV-2, heart failure, the pandemic, and coronavirus disease 2019. The lower left quadrant shows weakly developed, emerging, or declining themes and includes hydroxychloroquine, chloroquine, azithromycin, cardiac arrest, out-of-hospital cardiac arrest, and cardiopulmonary resuscitation.

### 4.9 Analysis of co-citation

Co-citation analysis is a crucial bibliometric method used to map the relationships between authors or documents, thereby revealing the intellectual structure of a research field. This technique identifies instances where two documents or authors are cited together by a third source, with nodes representing the co-cited documents and edges indicating the frequency of these co-citations. The size of each node reflects the number of times the document is cited, with larger nodes indicating higher citation frequency (20). Similarly, edge thickness represents the frequency of co-citation, with thicker edges indicating more frequent co-occurrences. The significance of each node is evaluated using two key metrics: Betweenness and Closeness. Betweenness measures how often a node acts as a bridge in the shortest path between other nodes, highlighting its role in the network. Closeness, calculated as the inverse of the sum of a node's distances to all other nodes, reflects the node's direct influence on the network (21).

**Figure 11:**
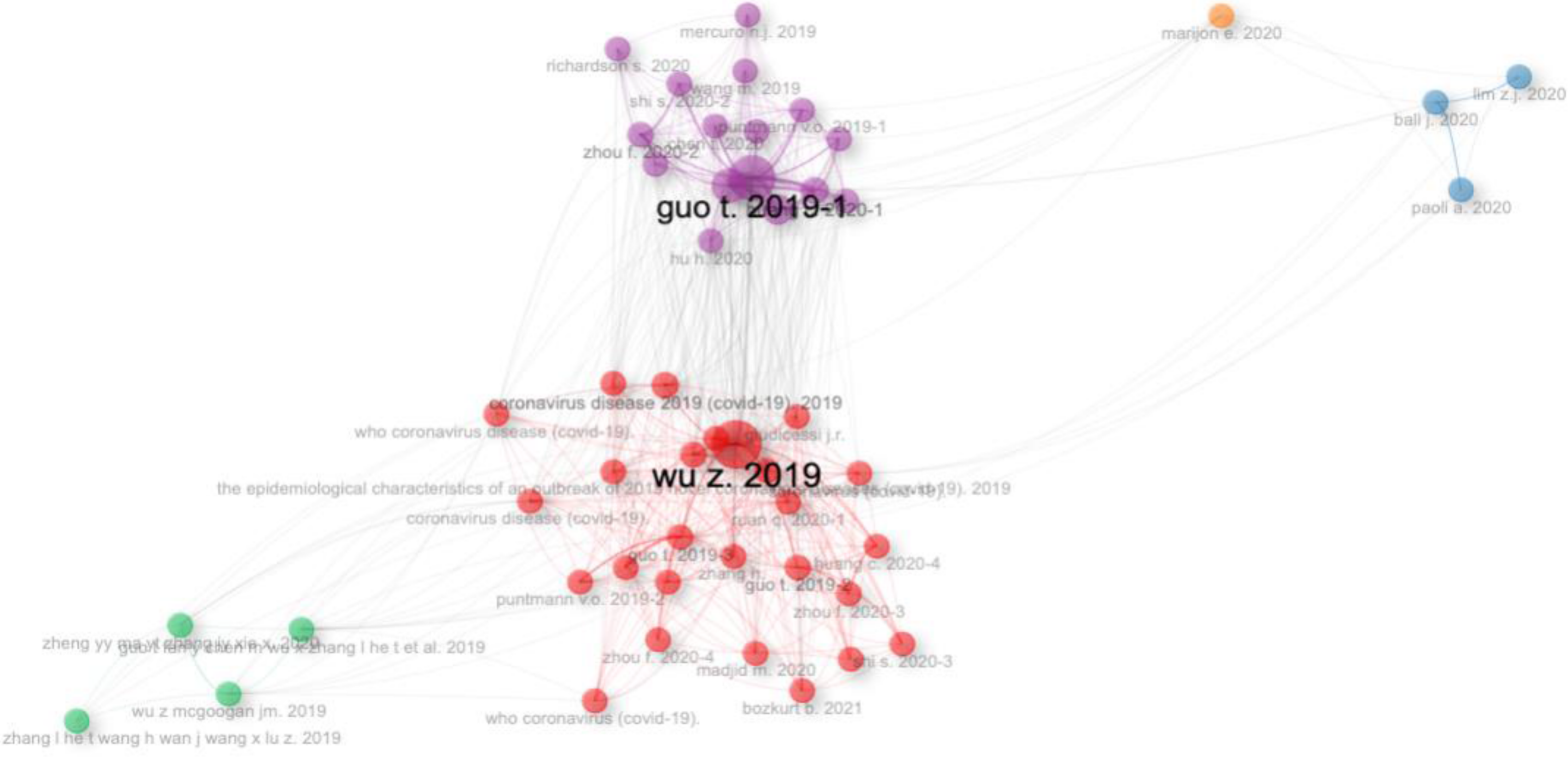
Co-citation network.

Figure 11 illustrates the research papers by Wu Z. 2019, Guo t. 2019–2, Guo t. 2019–3, Huang c. 2020–2, Lippi g. 2019, Ruan Q. 2020, puntmannv.o. 2019–2, Bozkurt b. 2021, Bonowr.o. 2019, Huang c. 2020–4, madjid m. 2020, zhou f. 2020–3, inciardir.m. 2019–2, puntmannv.o. 2019–3, giudicessij.r., zhou f. 2020–4, shi s. 2020–3, Varga z. 2020 and Zhang H.Have been extensively cited and co-cited by numerous documents within the dataset. It is represented by red colour in the Figure 11. Wu z mcgooganjm. 2019, guo t fan y chen m wu x zhang l he t et al. 2019, zhengyy ma ytzhangjyxie x. 2020, Guo t fan y Chen m Wu x Zhang l he t wang h wan j wang x lu z. 2019. Have also been co-cited by other source documents. It is represented by a purple colour in Figure 11. The colour of the nodes in the co-citation network represents the specific research field to which each record belongs.

### 4.10 Analysis of authors collaboration

**Figure 12:**
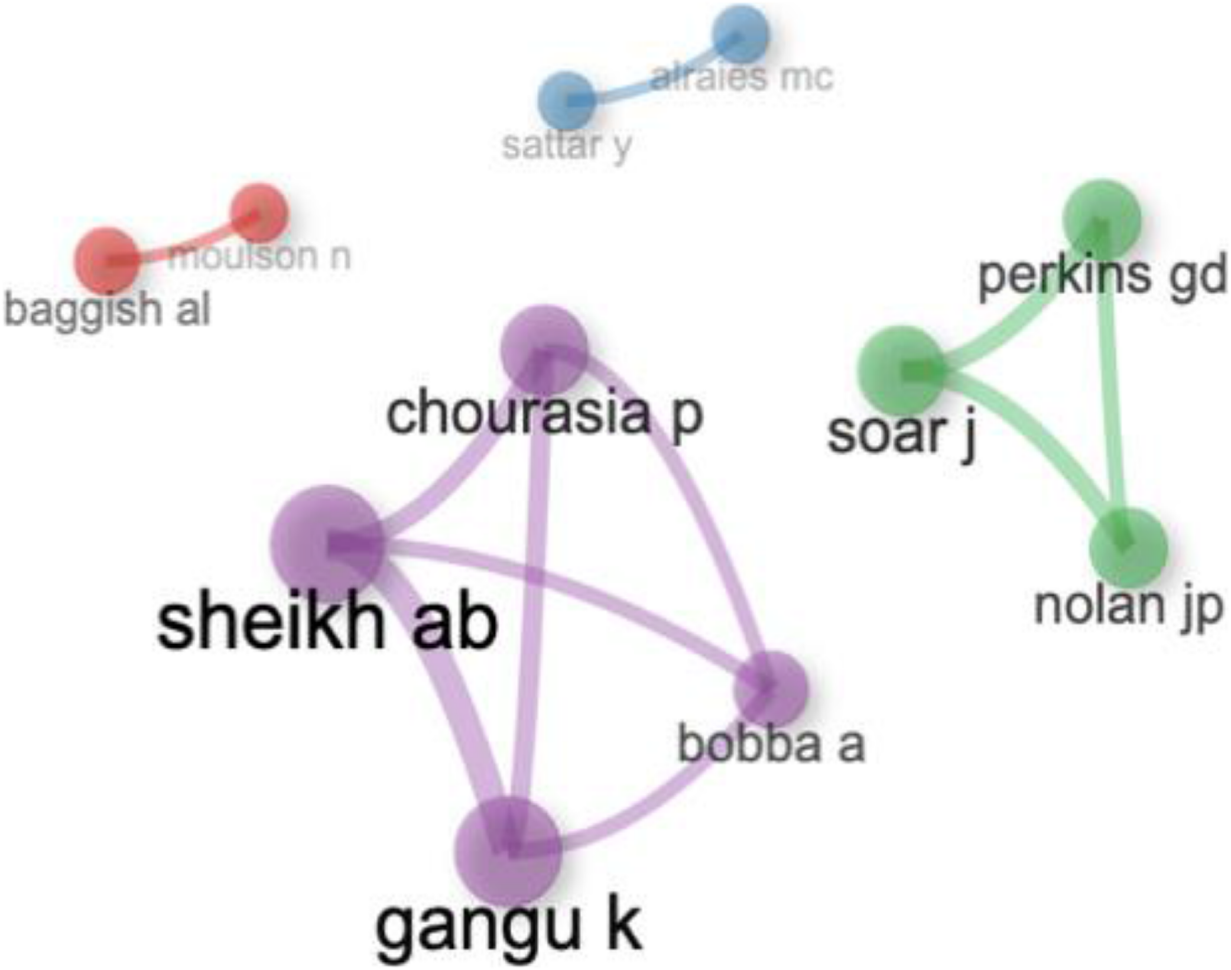
Author collaboration network.

The author's collaboration network analysis provides insight into how researchers interact and collaborate. We set a minimum threshold of four edges to map global author collaborations (22). Figure 12 illustrates the connections among the eight most prolific authors from our dataset. These eight authors, selected from a total of fifty, have shown significant collaboration, each with at least five publications. In the network, the thickness of the edges represents the strength of the collaboration between authors, while the size of each node reflects the number of co-authored articles (23). For example, in the purple-coloured segment, Bobb A., Chaurasia P., Gangu K., and Sheikh Ab are prominent for their high publication output, indicating strong collaboration within this group. Similarly, Sattar Y. and Alraies MC, depicted in the blue-coloured segment, also stand out for their substantial publication record. Lastly, alraies mc and sattar y, shown in the red segment, are noted for their extensive contribution to the literature. This analysis highlights the most active and collaborative researchers, showcasing their influential roles in the field.

### 4.11 Analysis of institutions

**Figure 13:**
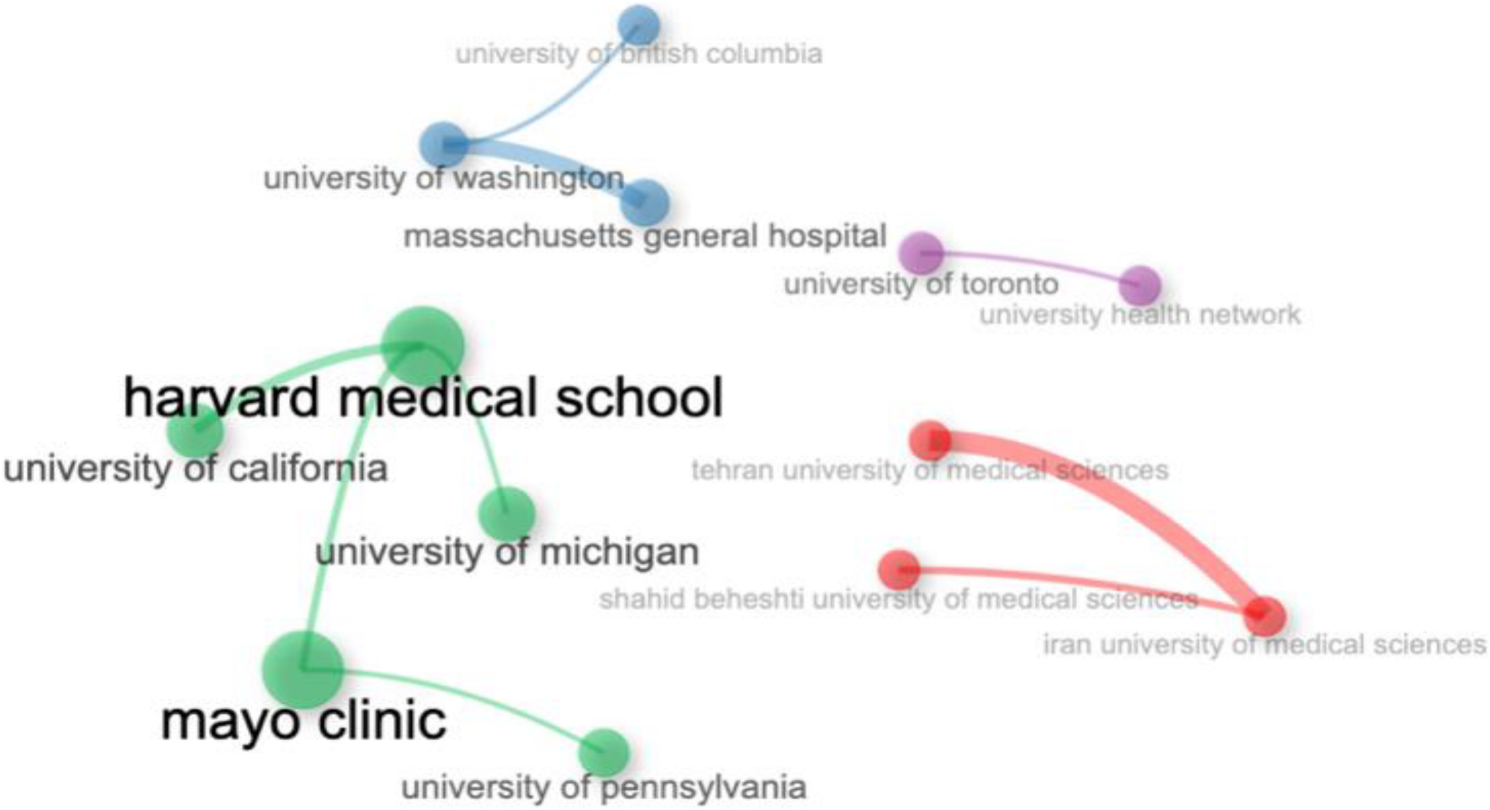
Institutional collaboration networks.

The analysis of institutional collaboration networks reveals the interactions and partnerships between various research institutions (24). We have mapped the global landscape of institutional collaborations by setting a threshold of five connections. In this network, the thickness of the edges signifies the strength of these collaborations, and the size of each node represents the volume of joint publications (25). Figure 13 highlights the top institutions from our dataset based on their collaborative activities. For instance, Harvard Medical School, the University of California, the University of Michigan, the Mayo Clinic, and the University of Pennsylvania are identified as leading contributors to collaborative research on COVID-19 and Sudden Cardiac Death.

Similarly, Iran University of Medical Sciences has frequently partnered with Tehran University of Medical Sciences and Shahid Beheshti University of Medical Sciences. The University of Washington has engaged in extensive collaborations with the University of British Columbia and Massachusetts General Hospital. Additionally, the University of Toronto has formed significant research partnerships with the University Health Network. This analysis underscores the key institutional players and their collaborative impact in these research fields (26).

### 4.12 Analysis of country

**Figure 14:**
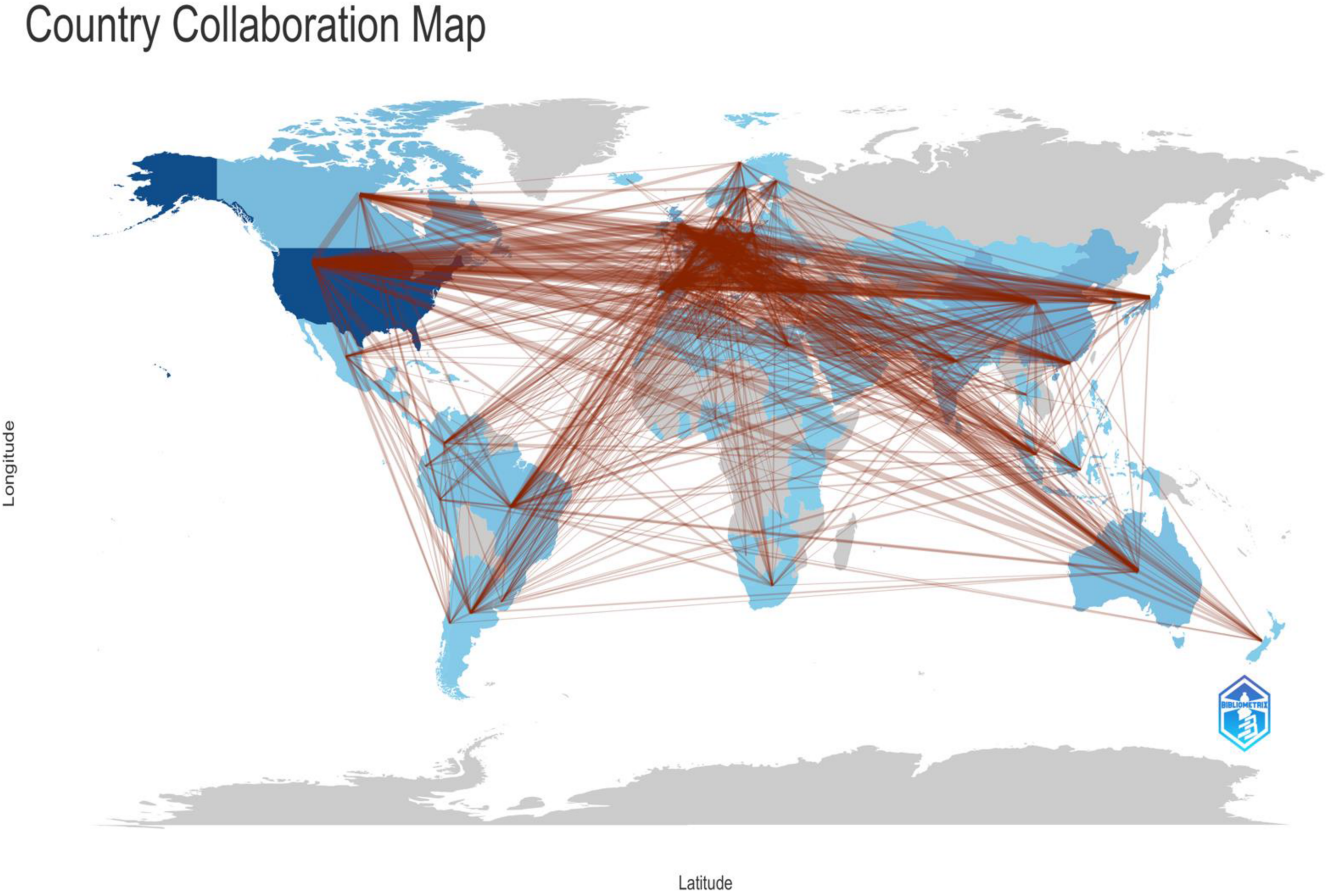
International collaboration Network.

Figure 14 depicts the international collaboration network, illustrating how countries interact and partner in research. We have mapped global collaborations in the COVID-19 and SCD domains by applying a threshold of five or more connections. According to Table 4, the United States has forged substantial collaborations with various countries, including 90 partnerships with the United Kingdom, 78 with Italy, 76 with Canada, 63 with Germany, and 52 with China. Italy, in turn, has established notable research collaborations with the U.K. (50 partnerships) and Germany (48 partnerships). This analysis highlights the prominent international research alliances and underscores the interconnected nature of global efforts in these research areas (26).

**Table 4:** International collaboration.

From	To	No of collaborations
USA	UNITED KINGDOM	90
USA	ITALY	78
USA	CANADA	76
USA	GERMANY	63
USA	CHINA	52
Italy	UNITED KINGDOM	50
Italy	GERMANY	48
USA	FRANCE	46
United kingdom	GERMANY	41
USA	AUSTRALIA	41

## 5 Discussion

The discussion of the bibliometric analysis on COVID-19 and Sudden Cardiac Death reveals several significant trends and patterns that offer valuable insights into the evolving research landscape in this field. The retrieval of 2,915 articles from SCOPUS between January 1, 2020, and August 8, 2024, underscores the rapid surge in scientific output related to these critical topics during the COVID-19 pandemic. The fact that the majority of these publications are original research articles, with reviews accounting for one fourth, suggests that there has been a strong focus on generating new empirical evidence and synthesizing existing knowledge to address the challenges posed by the intersection of COVID-19 and SCD. It was observed that not all 2,195 articles have “COVID-19” or “SCD” as a keyword it is explained below.

In bibliometric analysis, particularly when analysing authors’ keywords, it's common to see variations in keyword usage, even when articles focus on the same topic. As per the 2,195 articles searched under this literature review, only 1,363 occurrences of the keyword COVID-19 are highlighted. As the analysis of data through Biblioshiny is nomenclature specific, depending on the focus of the article, some authors have preferred “COVID-19” if they’re discussing the pandemic broadly, while others have used “SARS-CoV-2” and some other nomenclature related to COVID-19 while discussing the virus specifically but limiting words in the analysis up to 10 as per the number of occurrences (up to 99 occurrences displayed in figure) limits their inclusions in the figure added.

The peak in publications in 2021, with 842 articles, likely reflects the heightened global research activity as the pandemic unfolded, and its cardiovascular implications became more apparent. This surge in research output can be attributed to the urgent need to understand the mechanisms by which COVID-19 contributes to SCD, as well as to develop strategies for prevention and management. The subsequent decrease in the number of publications in the following years may indicate that the initial burst of research activity led to a saturation point, or it could reflect shifts in research priorities as the pandemic evolved. Interestingly, the citation analysis reveals a declining trend in the average number of citations per article over time, from 16.9 in 2020 to just 0.9 in 2024. This trend may be explained by several factors. The passage of time itself significantly affects citation patterns. Articles generally accumulate citations over time, meaning that more recent publications naturally have fewer citations simply because they have had less time to be read, referenced, and cited by other scholars. Older articles, on the other hand, have been available for a longer period, giving them more opportunities to be cited. Thus, the declining trend in the average number of citations per article from 2020 to 2024 could partly be explained by the shorter time window available for newer publications to gather citations. This temporal effect is common in citation analysis and should be considered when interpreting such trend.

The analysis of leading researchers, institutions, and countries highlights the concentrated nature of contributions to this field. Institutions like Harvard Medical School, Mayo Clinic, and the University of Pennsylvania are prominent, with Harvard leading the pack with 157 publications. The dominance of these institutions underscores their pivotal role in advancing research at the intersection of COVID-19 and SCD. Moreover, the United States, China, and the United Kingdom emerged as the top contributors, with the U.S. leading both in total publications and citations. The higher average citations per article for China suggest that research from this country has had a significant impact, possibly due to the high quality or relevance of the studies conducted (27, 28).

The co-occurrence and keyword analysis reveals key research themes, with “COVID-19” and “SARS-CoV-2” being the most frequently mentioned terms. The prominence of keywords like vaccination, mortality, troponin, biomarkers and cardiovascular events like myocarditis, out-of-hospital cardiac arrest, cardiac arrest, arrhythmia, cardiovascular disease, acute coronary syndrome, coronavirus, Takotsubo syndrome, and sudden cardiac death highlights the concerns associated with COVID-19 that have captured the attention of the research community. There are several study done that supports the results (29).

The initial dose of COVID-19 vaccines resulted in a general decrease in cardiovascular incidents, though rare cardiovascular complications were noted. A study by Samantha Ip et al. highlighted that the rates of common arterial thrombotic events, such as acute myocardial infarction and ischemic stroke, were typically reduced following each vaccine dose, regardless of brand or combination. Similarly, venous thrombotic events, including pulmonary embolism and deep vein thrombosis, occurred less frequently post-vaccination. However, there was an increase in the incidence of previously identified rare adverse effects after vaccination. Another study highlighted that those with higher levels of physical activity or superior cardiorespiratory fitness seem more resilient against the physical and mental health challenges posed by COVID-19 (9). While the study results by Samantha Ip et al. appeared to be a decrease in cardiovascular incidents following COVID-19 vaccination, this may not directly imply that the vaccines caused the decrease. The “healthy vaccine effect” (HVE) might explain the reduced hazard ratios, as the effect is most pronounced immediately after vaccination. Therefore, it is not necessarily accurate to attribute the decrease solely to the vaccine, as other factors like HVE could influence the observed trend (30).

Similarly, while much attention has been placed on radiological and laboratory investigations for early diagnosis and prognosis of COVID-19, it is essential to consider the broader picture. Much focus is on the radiological and laboratory investigation that can help in the early diagnosis and prognosis of COVID-19 disease. Cardiovascular Magnetic Resonance (CMR) has emerged as a crucial tool in diagnosing both ischemic and non-ischemic myocardial injuries, providing detailed insights into the impact of COVID-19 on myocardial tissue and function (28). The American College of Cardiology, the European Society of Cardiology, and the Society for CMR all emphasize that CMR is a useful diagnostic tool for patients with COVID-19 who show evidence of myocardial injury and cardiac dysfunction (28). Interestingly, the Chinese hospital data showed that 27.8% of admitted COVID-19 patients had a myocardial injury, with higher mortality rates in those with elevated troponin levels. However there is also medical literature explaining how SARS-CoV-2 infection may be not associated to clinical or biomarkers abnormalities (29). As an example, a retrospective study revealed that SARS-CoV-2 infection in elite soccer athletes was not linked to clinical or biomarker abnormalities. Elevated hs-cTnI levels were rare and not significantly associated with prior infection or pathological findings on CMR, though they were slightly more common in the infected group (29). There are still unsolved mysteries related to the troponin test, which is one of the important laboratory tests that need further research, and it can be a future point that needs to be clarified on how helpful it is for diagnostic purposes (29).

The clustering of these keywords into thematic maps provides a clear visualization of how different research topics within this field are interconnected and how they have evolved over time. For instance, well-developed themes such as COVID-19, SARS-CoV-2, and heart failure are crucial focal points of the research, while emerging or declining themes like hydroxychloroquine and cardiac arrest signal areas that may either need more exploration or have lost research momentum. The co-citation analysis further elucidates the intellectual structure of this research domain, identifying seminal works that have been extensively cited and co-cited, thereby shaping the discourse on COVID-19 and SCD. Articles such as those by Xu Z et al. and Guo T et al. have been highly influential, suggesting that they provide foundational knowledge or critical insights that are widely recognized within the research community (30). However, a mismatch between the top authors and the three-field plot (Source-Author-Keyword) in Biblioshiny has been observed. For example, author crea in Figure 4 seems to have a larger output than Chen Y. But Chen Y is not in Figure 3. It may be due to several reasons due to several reasons like.

The “Top authors” list is likely ranked by productivity (total number of publications), whereas the three-field plot might show relations based on co-occurrences or specific parameters. For instance, an author could be prolific but less associated with certain keywords or sources in the context of your dataset. Top authors may not always be the most frequently associated with the most common sources or keywords. The three-field plot reflects specific patterns between source, author, and keyword co-occurrence, while the “Top authors” list only shows the most productive authors, which might not align with the top sources or keywords. For example, a highly productive author might publish across multiple sources or use diverse keywords, which may not be as visible in a focused three-field plot. We have applied 20 as a limit for various parameters used. If the top authors aren't within that limit for the specific keyword or source, they might not appear in the plot, even though they are ranked high overall.

Finally, the network analyses of author, institutional, and international collaborations reveal the collaborative nature of research in this field. The strong partnerships among leading researchers and institutions indicate that collaboration is a key driver of high-impact research. The extensive international collaborations, particularly involving the United States, Italy, and the United Kingdom, reflect the global nature of the research efforts to tackle the challenges posed by COVID-19 and its cardiovascular implications (14, 29).

In conclusion, this bibliometric analysis demonstrates that the intersection of COVID-19 and SCD has been a major focus of global research since the onset of the pandemic. The trends in publication output, citation impact, and collaboration networks provide a comprehensive overview of the research landscape, highlighting key contributions, emerging themes, and potential areas for future investigation. This study not only maps the current state of research but also serves as a valuable resource for guiding future studies aimed at addressing the on-going challenges in understanding and mitigating the impact of COVID-19 on cardiovascular health. Additionally, research at the intersection of sudden cardiac death (SCD) and COVID-19 highlights the complex relationship between the virus and cardiovascular health. COVID-19 has been shown to exacerbate underlying heart conditions, increasing the risk of SCD in vulnerable populations, particularly those with pre-existing cardiovascular diseases, cardiomyopathies, and channelopathies (14, 29). The pro-inflammatory and pro-thrombotic environment caused by COVID-19, along with its potential to trigger arrhythmias, contributes to an elevated risk of fatal cardiac events. This emerging evidence underscores the need for heightened surveillance and targeted interventions in high-risk individuals during the pandemic, as well as continued research to better understand the mechanisms driving SCD in the context of COVID-19 (14, 29).

Future research avenues in the intersection of COVID-19 and SCD should focus on several key areas to deepen our understanding and improve clinical outcomes. First, there is a need for longitudinal studies to investigate the long-term cardiovascular effects of COVID-19, particularly in patients who have recovered but remain at risk for sudden cardiac events. Research should also explore the underlying mechanisms by which COVID-19 exacerbates pre-existing cardiovascular conditions, potentially leading to sudden cardiac death. Additionally, the role of genetic predispositions and biomarkers in identifying high-risk individuals warrants further investigation. Another promising avenue is the development and evaluation of targeted therapies and interventions to mitigate the cardiovascular impacts of COVID-19, especially in vulnerable populations. Finally, expanding global collaboration and data sharing can enhance the generalizability of findings and foster a more comprehensive approach to addressing these complex health challenges.

## Data Availability

The original contributions presented in the study are included in the article/Supplementary Material, further inquiries can be directed to the corresponding author.
